# A Novel Salicylaldehyde Dehydrogenase from Alpine Soil Metagenome Reveals a Unique Catalytic Mechanism

**DOI:** 10.1007/s12010-025-05445-4

**Published:** 2025-11-03

**Authors:** Shamsudeen Umar Dandare, Ibrahim Aliyu Dabai, Deepak Kumaresan, Christopher C. R. Allen

**Affiliations:** 1 School of Biological Sciences, Queen’s University Belfast, Belfast, BT9 5DL UK; 2 Department of Biochemistry and Molecular Biology, Usmanu Danfodiyo University, Sokoto, Nigeria; 3 Agri-Environment Branch, Agri-Food and Biosciences Institute, Belfast, BT9 5PX UK; 4 School of Biosciences, University of Birmingham, Birmingham, B15 2TT UK

**Keywords:** Metagenomics, Enzyme kinetics, Biocatalysis, Molecular docking, Phylogenetic analysis, Environmental biotechnology

## Abstract

Metagenomic approaches have revolutionised the discovery of novel enzymes with ecological and biotechnological significance from different environments. Here, we report the comprehensive characterisation of a novel salicylaldehyde dehydrogenase (SALD_AP_) obtained from an alpine soil metagenome. Phylogenetic analysis revealed that SALD_AP_ is the first experimentally characterised Alphaproteobacterial SALD, forming a distinct evolutionary clade among known bacterial enzymes. The recombinant enzyme exhibited strict specificity for NAD⁺ and exceptional catalytic efficiency toward aromatic aldehydes, with benzaldehyde as the preferred substrate. Kinetic analyses showed catalytic efficiencies exceeding 10⁶ M⁻¹ s⁻¹ for aromatics, whereas aliphatics were oxidised with much lower efficiency, consistent with ecological specialisation for aromatic catabolism in alpine soils enriched in lignin-derived compounds. SALD_AP_ was most active under mildly alkaline conditions (optimum pH 8.0) and tolerated a range of chemical environments, though high concentrations of certain metals and solvents were inhibitory. Differential scanning fluorimetry demonstrated that the enzyme was stabilised by ligand binding, with maximal thermal stability observed when both substrate and cofactor were present. Structural alignment with *Pseudomonas* NahF and docking analyses revealed that SALD_AP_ employs a distinctive catalytic configuration involving ASN-137, ARG-145, GLU-238, and CYS-272, highlighting a non-canonical role for ASN-137 in substrate binding and stabilisation. Based on these findings, we propose a mechanistic model for SALD_AP_ that expands the catalytic diversity of the aldehyde dehydrogenase superfamily. This study establishes a new paradigm for aromatic aldehyde oxidation, underscores the ecological significance of SALD_AP_ in alpine soil microbiomes, and provides a foundation for engineering novel biocatalysts for bioremediation and synthetic biology applications.

**Supplementary Information:**

The online version contains supplementary material available at 10.1007/s12010-025-05445-4.

## Introduction

Polycyclic aromatic hydrocarbons (PAHs) and their derivatives are common environmental pollutants and metabolic intermediates that require complete microbial degradation to prevent accumulation in soils and aquatic systems. In bacteria, PAH degradation typically proceeds through a series of enzymatic steps broadly divided into “upper” and “lower” pathways. The upper pathway involves the oxidation of hydrocarbons (such as naphthalene, pyrene, phenanthrene) to catechols and salicylaldehydes, whereas the lower pathway converts these intermediates, particularly salicylate, into tricarboxylic acid (TCA) cycle intermediates through meta- or ortho-cleavage routes, completing the mineralisation of the original PAH [1–3]. Salicylaldehyde dehydrogenases (SALDs; EC 1.2.1.65) occupy a central position in this network by catalysing the NAD(P)⁺-dependent oxidation of salicylaldehyde to salicylate, a critical step linking hydrocarbon oxidation with ring-cleavage metabolism [4, 5].

SALDs belong to the aldehyde dehydrogenase (ALDH) superfamily, one of the most structurally and functionally diverse enzyme groups in biology. Members of this superfamily are distributed across all domains of life and catalyse the oxidation of a wide range of aliphatic and aromatic aldehydes, contributing to detoxification, primary metabolism, and specialised catabolism [6, 7]. Despite their conserved Rossmann-fold nucleotide-binding domain and an invariant catalytic cysteine, ALDHs exhibit remarkable diversity in oligomeric state (dimers, tetramers, hexamers), cofactor preference (NAD⁺ vs. NADP⁺), and substrate specificity [6, 8, 9]. Recent structural and kinetic studies have highlighted how subtle changes in active-site residues or cofactor-binding loops can markedly influence enzyme activity, stability, and substrate range [10, 11].

Despite their central role in aromatic hydrocarbon metabolism, SALDs remain underexplored compared with other ALDHs. To date, only a handful have been structurally or biochemically characterised, mostly from *Pseudomonas* and related genera. The in vivo activity of SALDs has been reported in several PAH-degrading microorganisms [12–16], but detailed in vitro biochemical analyses remain limited. Zhao and colleagues [17] purified and partially characterised two SALDs (NahV and NahF) from *P. putida* ND6. Additional studies have examined SALDs from *Pseudomonas* sp. strain C6 [18] and *Alteromonas naphthalenivorans* [4]. Notably, Coitinho and colleagues reported the first crystal structure and kinetic analysis of a broad-substrate SALD (NahF) from *P. putida* G7 [1]. We recently reported the crystallographic structure of the first metagenome-derived salicylaldehyde dehydrogenase (SALD_AP_) from alpine soil [19], which shares only 43% amino acid identity with the well-characterised NahF from *P. putida* G7, highlighting the sequence diversity within this enzyme family. That work provided the first structural description of a metagenome-derived SALD but did not address its biochemical, kinetic, or mechanistic properties. Building on this structural report, the present study provides the first comprehensive biochemical and mechanistic characterisation of SALD_AP_, integrating phylogenetic analysis, substrate profiling, steady-state kinetics, differential scanning fluorimetry, and docking-guided mechanistic analyses.

A major novelty of this work lies in the phylogenetic placement of SALD_AP_. Whereas previously studied SALDs originate mainly from Beta- and Gammaproteobacteria, SALD_AP_ represents the first experimentally characterised Alphaproteobacterial SALD. Its discovery and functional analysis expand the phylogenetic and mechanistic breadth of the SALD family and offer new insights into cofactor specificity and catalytic strategy.

The alpine soil origin of SALD_AP_ further underscores its ecological and biotechnological relevance. Alpine ecosystems are characterised by nutrient-poor and cold-adapted conditions, with microbial communities that play crucial roles in the turnover of plant- and pollutant-derived aromatics. Enzymes from such environments often exhibit distinctive adaptations, such as stability under stress and activity on structurally demanding substrates, making them valuable candidates for biocatalysis and synthetic biology [20–22].

In this study, we report the comprehensive characterisation of SALD_AP_, a novel salicylaldehyde dehydrogenase identified from an alpine soil metagenome. Through integrated phylogenetic, structural, kinetic, ligand-binding, and molecular docking analyses, we show that SALD_AP_ is the first experimentally characterised Alphaproteobacterial SALD and exhibits a unique catalytic mechanism involving a strictly conserved asparagine residue. SALD_AP_ displays strict NAD⁺ dependence, high catalytic efficiency for aromatic aldehydes, and is stabilised by substrate and cofactor. These findings expand the functional and mechanistic landscape of the ALDH superfamily and provide a foundation for engineering novel biocatalysts for environmental and synthetic biology applications.

## Materials and Methods

### Soil Sample Collection and Metagenomic Library Construction

This procedure has been described previously [22]. In brief, soil samples were aseptically collected at mid-depth from different horizons (Ah, Bw, Cox, Cu) in glacial moraines from the Guil and Po River valleys of the French and Italian Alps, respectively. Samples were transported on ice and stored at −20 °C. Total DNA was extracted using the PowerSoil DNA extraction kit (QIAGEN) according to the manufacturer’s instructions, with minor modifications. DNA concentrations were determined using a Quantus Fluorometer (Promega). DNA libraries were prepared and sequenced in paired-end mode on an Illumina MiSeq at the University of Cambridge DNA sequencing facility. Raw metagenome data are available in the NCBI Sequence Read Archive (Bioproject number: PRJNA490486).

### Gene Mining, Assembly, and Phylogenetic Analysis

To identify aldehyde dehydrogenases (ALDHs), a vanillin dehydrogenase from *Pseudomonas putida* KT2440 (NP_745497.1) served as a reference for BLAST searches against merged Illumina datasets. Sequence data from different soil horizons and nearby sample sites were combined to maximise coverage. Discontinuous MagaBLAST (dc-megablast) was used with an E-value threshold of 1.0e-5 and a minimum identity of 70%. Hit sequences were extracted and used as seeds for gene-targeted assembly using the PRICE *de novo* assembler [23], which builds longer contigs from paired-end reads. PRICE was run for ten assembly cycles, with only contigs matching initial BLAST hits retained. Contigs longer than the expected gene length were analysed for open reading frames (ORFs) with NCBI’s ORF Finder (bacterial code). The quality and novelty of each ORF were confirmed by BLAST. The assembled SALD_AP_ sequence is available in NCBI with the GenBank accession PV600639.

Protein sequences were aligned with Clustal Omega [24], and poorly aligned regions were trimmed with trimAl [25]. A phylogenetic tree was constructed using FastTree [26], and visualised in iTOL [27].

### Cloning, Expression, and Purification of SALD_AP_

Primers designed based on the assembled SALD_AP_ sequence were used to amplify the gene from alpine soil metagenomic DNA. The PCR product was cloned into the pLATE51 vector and expressed as previously described [19]. The recombinant plasmid (pLATE51-SALD_AP_) was transformed into chemically competent *E. coli* BL21 (DE3) cells. Cultures were grown to OD₆₀₀ ≈ 0.6 at 30 °C, induced with 1 mM IPTG, and incubated for 6 h. Cells were harvested by centrifugation (5,000 × g, 10 min, 4 °C) and resuspended in lysis buffer (50 mM sodium phosphate, 300 mM NaCl, 5 mM imidazole, pH 8.0) supplemented with 0.2 mg mL⁻¹ lysozyme and 0.5 mM phenylmethylsulfonyl fluoride (PMSF). Following 15 min incubation on ice, cells were disrupted by sonication on ice (three 30 s pulses at 16 μm amplitude, with 30 s cooling intervals). The soluble fraction was obtained by centrifugation (13,000 × g, 30 min, 4 °C) and purified by metal-affinity chromatography using gravity flow with a 1 mL His-Select cobalt resin (Sigma-Aldrich) packed into Econo columns (Bio-Rad), which were equilibrated with the lysis buffer. Bound protein was washed with buffer identical to the lysis buffer except containing 10 mM imidazole and eluted with the same buffer containing 250 mM imidazole. The eluate was buffer-exchanged and concentrated at 4 °C against 50 mM sodium phosphate, pH 8.0, 150 mM NaCl, and 1 mM DTT using an Amicon Ultra-15 centrifugal filter unit with a 30 kDa molecular weight cutoff (Merck Millipore, Ireland). The recombinant protein was expressed with an N-terminal 6×His tag, and all experiments were performed with the purified 6×His-SALD_AP_.

### Enzyme Activity Assay

The activity of purified SALD_AP_ was measured as described by Coitinho and colleagues [1] with modifications. All assays were performed at 25 °C in a final volume of 1 mL using a 6705 UV-Vis spectrophotometer (Jenway) with a 1 cm pathlength quartz cuvette. The assay was done to monitor NAD⁺ reduction to NADH at 340 nm. The reaction mixture contained 200 µM salicylaldehyde (SAL), 200 µM NAD⁺, and 1–2 µM purified enzyme in 1 mL of sodium phosphate buffer (50 mM, pH 8.0). Control reactions without the enzyme were included as blanks. Absorbance at 340 nm was recorded continuously for 10 min, and initial rates (v) were determined from the linear portion of the progress curve by linear regression. Rates were converted to molar units using NADH extinction coefficient (ε₃₄₀ = 6,220 M⁻¹ cm⁻¹) [28]. Enzyme activity was expressed in units (U), where 1 U corresponds to the formation of 1 µmol NADH per minute (1 µmol min^− 1^). All assays were performed in triplicate, and results are reported as mean ± standard deviation (SD). Formation of salicylate from salicylaldehyde was additionally confirmed using the salicylate colourimetric assay kit (Sigma-Aldrich) as described by the manufacturer.

### pH Profile and Thermostability of SALD_AP_

The pH optimum was determined by measuring activity across pH 5.3–11.1 using appropriate buffers: Bis-Tris (pH 5.3–6.3), HEPES (pH 6.7–7.5), Tricine (pH 7.4–8.8) and CAPS (pH 9.8–11.1). Assays at each pH were run in triplicate using a Multiskan™ microplate spectrophotometer. Rates were normalised to the highest activity, expressed as a percentage of the maximum activity. Thermostability was assessed by incubating 1 µM enzyme in sodium phosphate buffer (50 mM, pH 8.0) at temperatures ranging from 40 to 60 °C using a heating block. Aliquots were withdrawn at 10 min intervals for up to 60 min, cooled on ice, and residual activity was determined under standard assay conditions.

### Effect of Compounds on Enzyme Activity

Enzyme activity was tested in the presence of various additives. A 1 µM 6×His-SALD_AP_ was pre-incubated at 4 °C for 15 min in Tricine buffer (100 mM, pH 8.0) containing the additive. Tested compounds included metal ions (up to 50 mM), detergents (Tween-20 and Triton X-100 at 1–10% w/v), DTT (0.5–50 mM), EDTA (10–500 µM), and organic solvents (methanol, ethanol, isopropanol, and DMSO at 1–50% v/v). Following pre-incubation, salicylaldehyde (70 µM) and NAD⁺ (50 µM) were added to start the reaction, and activity was measured. All measurements were performed in triplicate and are reported as mean ± standard deviation (SD). Enzyme activity was expressed as a percentage of the control (no additive).

### Substrate Specificity and Steady-State Kinetics

Optimal NAD⁺ and enzyme concentrations were established by first varying NAD⁺ concentrations (0–1000 µM) with 1 µM enzyme and 200 µM salicylaldehyde, then varying enzyme (0–1 µM) at the optimal NAD^+^ concentration. The enzyme concentration was varied to identify a range that provided linear initial rates while avoiding substrate depletion or limitations due to cofactor availability. Protein concentration was determined using the Bradford assay with bovine serum albumin (BSA) as a standard. Steady-state kinetics were performed at 25 °C with 50 µM NAD⁺ and varying substrate (2–1500 µM) in 100 mM Tricine buffer (pH 8.0, 1 mL volume). All assays contained 1.7% (v/v) DMSO to ensure consistency across measurements. This concentration was used to aid solubility for phenolic and long-chain (C7–C10) aldehydes and has been reported to have minimal effect on ALDH activity [29, 30]. Moreover, low concentrations of DMSO (≤ 3%) have been shown to exert a protective effect on the quaternary structure of some proteins and their interactions [31]. Initial rates were calculated as described above. Kinetic parameters (*Kₘ*,* Ki*,* Vₘₐₓ*) were obtained by fitting rate data to the Michaelis-Menten and substrate inhibition models (Eqs. 1 and 2) using GraphPad Prism 8.0. Turnover number (*k*_*cat*_) was calculated using Eq. 3 below, where [Eo] represents the total enzyme concentration [32, 33].

1 $$\:v=\frac{V_{max}\left[S\right]}{K_m+\left[S\right]}$$

2 $$\:v=\frac{V_{max}\left[S\right]}{K_m+\left[S\right]+\frac{\left[S\right]^2}{K_i}}$$

3 $$\:k_{cat}=\frac{V_{max}}{{\left[E\right]}_o}$$

Where *V*_*max*_ is the maximum velocity, *k*_*m*_ is the Michaelis-Menten constant, *K*_*i*_ is the substrate inhibition constant, and [*S*] is the substrate concentration.

### Differential Scanning Fluorimetry (DSF)

DSF was performed with SYPRO Orange dye to assess thermal stability and ligand effects. Enzyme (5–7 µM) in 50 mM HEPES buffer (pH 7.4) was mixed with substrates, NAD⁺, and Ca²⁺ as required. Substrates were prepared in DMSO and kept at a concentration below 1%. SYPRO Orange was added immediately before measurement. Triplicate reactions were performed in 0.2 mL PCR tubes using a Rotor-Gene Q cycler (Qiagen), with a temperature ramp from 25 °C to 95 °C at 1 °C/s. Fluorescence (excitation 460 nm, emission 510 nm) was used to monitor protein unfolding. The melting temperature (Tₘ) was determined from the first derivative of the fluorescence (ΔF/ΔT).

### Molecular Docking

Docking was performed with AutoDock Vina 1.1.2 with UCSF Chimera 1.12 as the interface. Crystal structures of SALD_AP_ (PDB ID: 6QHN) and *P. putida* G7 NahF (PDB ID: 4JZ6) were prepared by removing non-standard residues and ligands, adding hydrogens, and assigning Gasteiger charges. Ligands were built in Chimera from PubChem SMILES, energy-minimised, and converted to.pdbqt format. Docking used grid boxes centred on residues within 6 Å of the co-crystallised ligands. Default parameters were used for docking. Binding interactions and hydrogen bonds were visualised in Chimera and PyMOL 2.6. Dissociation constants (K_d_) were calculated from binding affinities (ΔG) using:

4 $$K_d=e^{\left(\frac{\bigtriangleup G}{RT}\right)}$$

where R is the gas constant (1.987 cal mol^− 1^ K^− 1^) and T is the temperature (298.15 K) [34].

### Statistical Analysis

All data are presented as mean ± standard deviation (SD) from at least three independent measurements. Statistical analyses were carried out using GraphPad Prism version 8.0 (GraphPad Software, San Diego, CA, USA). For datasets involving comparisons between treatments and a single control (e.g., thermal shift assays), one-way analysis of variance (ANOVA) followed by Dunnett’s multiple comparison post-test was applied. Statistical significance was defined at *p* < 0.05. Significant differences are indicated in the tables with an asterisk (*).

## Results and Discussion

### Sequence and Phylogenetic Analysis of Assembled Salicylaldehyde Dehydrogenase (SALD_AP_)

After ten assembly cycles, 215 contigs were recovered, with only five exceeding 1,000 nucleotides. Analysis revealed two major ORFs (Table S1), and only the second contig encoded a full-length gene with both start and stop codons. BLAST analysis revealed that this gene shares only 82% nucleotide identity with known sequences in NCBI and contains an aldehyde dehydrogenase superfamily (ALDH-SF) and a salicylaldehyde dehydrogenase DoxF-like (ALDH SaliADH) domain (Figure S1), confirming it as a bona fide SALD enzyme.

**Fig. 1:**
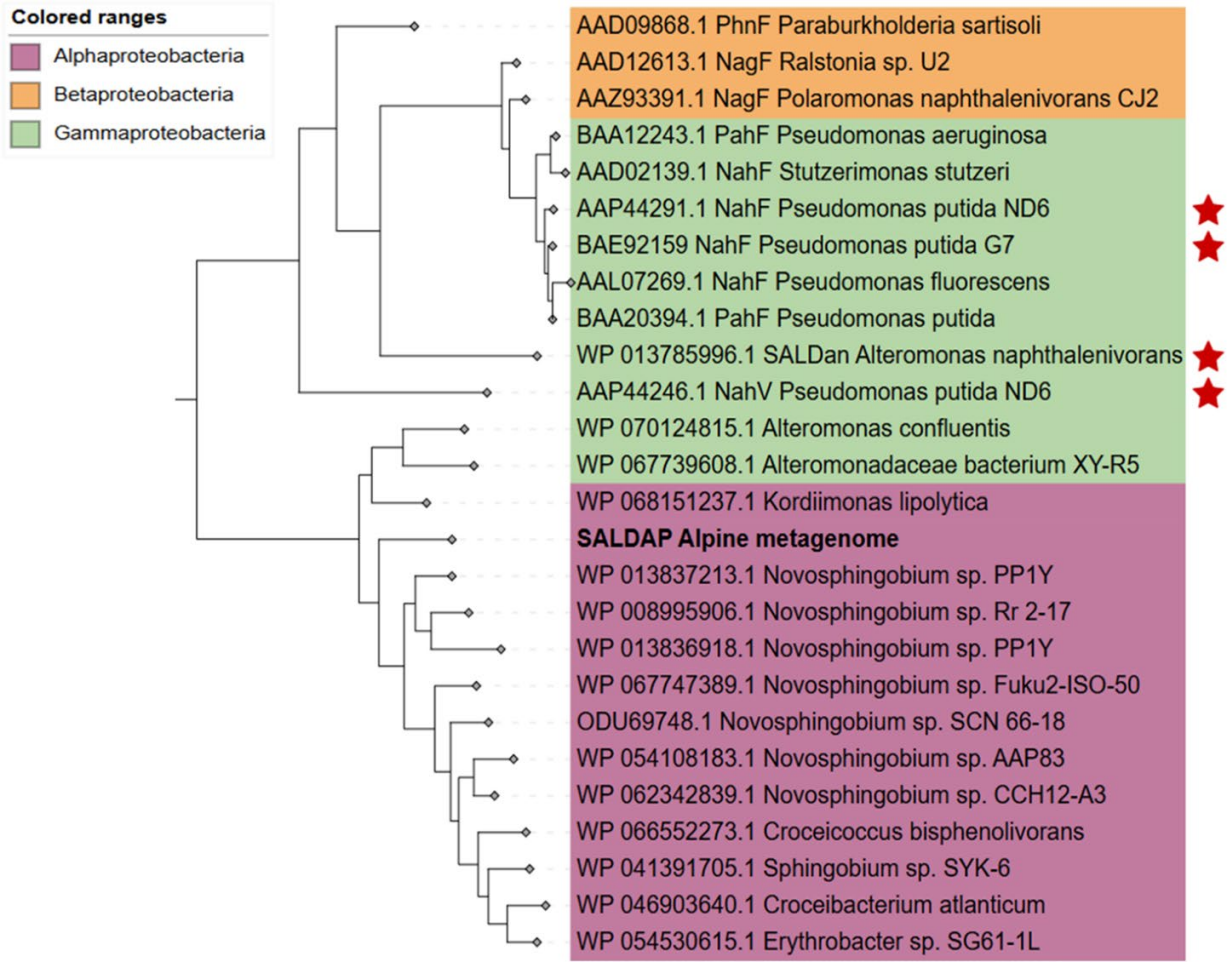
Phylogenetic relationship of metagenome-derived SALD_AP_ (bold letters) and other bacterial salicylaldehyde dehydrogenases (465–483 amino acids). All sequences included are annotated amino acid sequences retrieved from the NCBI protein database. The tree was computed using the maximum likelihood method (with 1000 bootstrap replicates) and the Jones-Taylor-Thornton (JTT) matrix-based model for calculating evolutionary distances. The tree was drawn to scale with branch lengths measured in the number of substitutions per site. Entries marked with red stars correspond to enzymes that have been biochemically characterised and shown to possess activity, whereas the remaining entries represent predicted proteins without experimental validation

Phylogenetic analysis placed SALD_AP_ within a distinct Alphaproteobacteria cluster (including *Croceicoccus*,* Erythrobacter*,* Kordiimonas* and *Novosphingobium*), separate from clusters of Beta- (*Ralstonia*,* Paraburkholderia* and *Polaromonas*) and Gammaproteobacteria (*Pseudomonas* and *Alteromonas*) (Fig. 1). Importantly, the related sequences used in this analysis are annotated amino acid sequences predicted from genomic data, with no reported biochemical or functional characterisation. This underscores the novelty of SALD_AP_ as the first biochemically characterised alphaproteobacterial SALD. Previous survey [4] showed most bacterial SALDs are distributed among *Betaproteobacteria* (31.33%), *Gammaproteobacteria* (21.58%), and *Alphaproteobacteria* (16.80%); however, to our knowledge, no alphaproteobacterial SALD has yet been characterised, motivating our study.

**Fig. 2:**
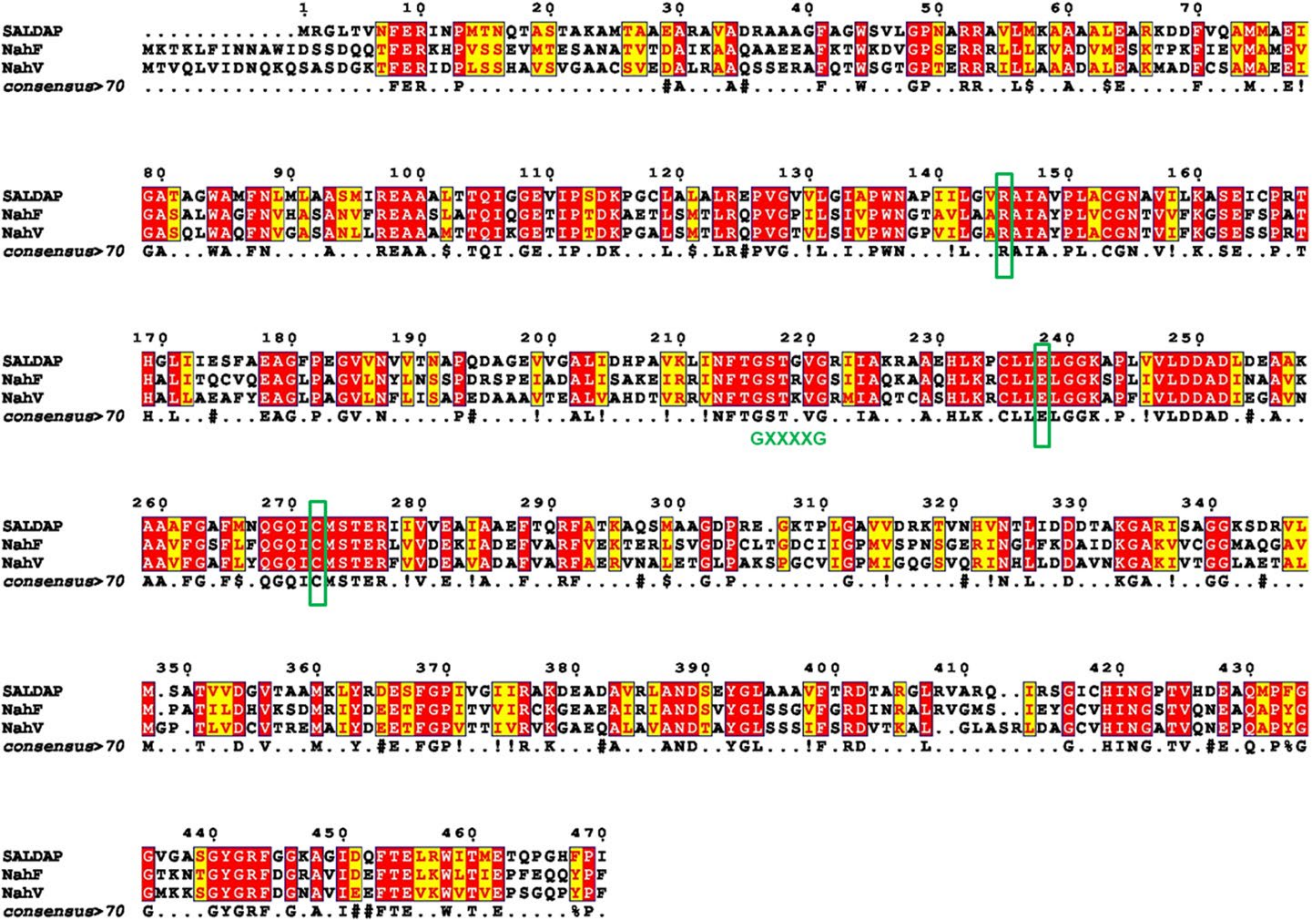
Multiple sequence alignment (MSA) of novel SALD_AP_ with class 2 ALDHs. NahF: Salicylaldehyde dehydrogenase from *Pseudomonas putida* G7 and NahV: Salicylaldehyde dehydrogenase from *Pseudomonas putida* ND6. Identical residues are boxed in red, while similar residues are in yellow. Green rectangles represent critical conserved residues in the catalytic domain. The glycine-rich Rossmann fold motif (GXXXXG) is shown in green. Notably, SALD_AP_ contains a glycine at the position where NahF has a conserved arginine implicated in stabilising the 2′-phosphate of NADP⁺, providing a structural basis for the strict NAD⁺ preference by SALD_AP_

Multiple sequence alignment revealed that SALD_AP_, NahF, and NahV share 40% amino acid identity, with key conservation observed in their catalytic and cofactor-binding domains. Notably, the cofactor binding domain, which conforms to the Rossmann fold (residues 128–221) contains a glycine-rich motif (GSTXVG, residues 216–221), analogous to the G_1_XXXXG_2_ motif in other NAD^+^-dependent dehydrogenases (Fig. 2) [8, 35]. The invariant catalytic cysteine (Cys272), as well as substrate-binding residues Asn137, Arg145, Glu238 and Leu239, are conserved. Aromatic residues, which may be implicated in substrate recognition (Trp83, Phe87 and Phe262) [1], are also preserved.

### Isolation of SALD_AP_ from Soil Metagenome and Recombinant Production

Specific primers designed from the SALD_AP_ sequence enabled its targeted amplification from alpine metagenomic DNA (Figure S2a), where it was present in all samples tested. The gene was cloned and optimally expressed, yielding a soluble protein that was purified to homogeneity using a Co^2+^-affinity resin (Figure S2b). From 1 L of culture, approximately 2 mg/mL (40 µM) purified protein was obtained. SDS-PAGE analysis indicated a monomeric molecular weight of 49.6 kDa, determined by comparing the relative migration of SALD_AP_ with the protein size markers (Figure S2c). However, SDS-PAGE is a denaturing technique and does not reflect the native oligomeric state of the enzyme. Our previous crystallographic and analytical gel-filtration studies demonstrated that SALD_AP_ exists as a homodimer in solution, with a molecular mass of 115 kDa, consistent with its theoretical dimeric size (110 kDa) [19]. This dimeric assembly is characteristic of class 3 aldehyde dehydrogenases such as vanillin and benzaldehyde dehydrogenases, and contrasts with the tetrameric (dimer-of-dimers) assemblies observed in class 1 and 2 ALDHs [36]. The oligomeric nature of SALD_AP_ therefore aligns well with its structural classification within the ALDH superfamily.

### SALD_AP_ Is a NAD^+^-dependent Aldehyde Dehydrogenase

The purified enzyme’s activity was assayed using SAL as substrate, with NAD^+^ and NADP^+^ as possible cofactors. SALD_AP_ showed strong activity with NAD^+^ but none with NADP^+^, confirming that it is a strictly NAD^+^-dependent ALDH. This selectivity reflects a common pattern among ALDHs, where NAD^+^ is typically used in oxidative degradation, while NADP^+^ is reserved for reductive biosynthesis [37].

Sequence comparison of the nucleotide-binding site revealed a key difference between SALD_AP_ and the well-characterised *Pseudomonas putida* NahF, which can utilise both NAD⁺ and NADP⁺ [1]. In NahF, the binding pocket contains a conserved arginine that stabilises the 2′-phosphate of NADP⁺, whereas in SALD_AP_ this residue is replaced by glycine (Fig. 2). This structural difference is highlighted in Fig. 2, where the Rossmann fold motif (GXXXXG) is conserved across ALDHs, but the glycine substitution in SALD_AP_ provides a plausible explanation for its strict NAD⁺ dependence. The absence of this positively charged side chain likely contributes to the exclusion of NADP⁺. However, broader structural analyses of NAD(P)⁺–protein complexes indicate that cofactor selectivity arises not from a single residue but from the overall architecture of the Rossmann fold, particularly the glycine-rich G1XXXXG2 motif [37]. In SALD_AP_, this motif (GSTGVG, residues 228–233) is conserved and well-suited for NAD⁺ binding, which explains its strict preference for NAD⁺ as a cofactor.

### SALD_AP_ Has an Alkaline pH Optimum and Is Stable Over a Wide Temperature Range

**Fig. 3:**
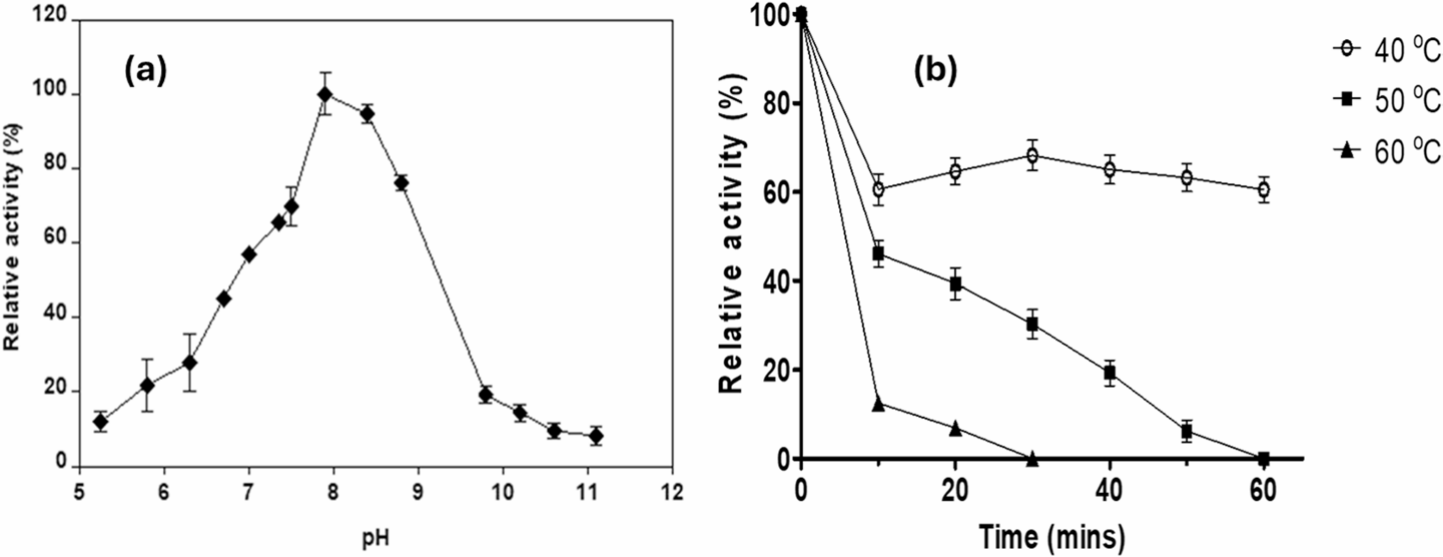
(**a**) Effect of pH on the activity of 6xHis-SALD_AP_ for the oxidation of salicylaldehyde at 25 °C, in the presence of 200 µM NAD^+^. Activities are reported as values relative to the activity of 1 µM protein (set as 100%) at pH 8.0. Values represent the means of triplicate measurements (mean ± standard deviation). (**b**) Relative activity of SALD_AP_ under standard assay conditions after preincubation of the enzyme at different times. Data points are the average of triplicate measurements; error bars represent ± SD

The optimal pH for recombinant 6xHis-SALD_AP_ was pH 8.0, with a bell-shaped activity profile across pH 5.3–11.1. Activity dropped by over 30% with a one-unit shift from the optimum, indicating high sensitivity to pH changes. At least 40% and 35% activity loss was observed at pH 7.0 and 9.0, respectively (Fig. 3a). This behaviour is consistent with classical SALDs from *Pseudomonas* species [1, 18] and other reported ALDHs [38–40].

Thermostability studies showed that 6xHis-SALD_AP_ retained more than 60% activity after 1 h at 40 °C, but was quickly inactivated at higher temperatures, where it exhibited marginal activity at 50 °C after 1 h and complete inactivation after 30 min at 60 °C (Fig. 3b). Compared to NahF from *P. putida*, which remains active at elevated temperatures for a longer period [1], SALD_AP_ is less thermostable but remains robust under moderate conditions.

### Effect of Metal Ions, Organic Solvents, Detergents, and Inhibitors on SALD_AP_ Activity

The activity of SALD_AP_ was affected by several metal ions, solvents, detergents, and inhibitors. At 1 mM, Fe^2+^ and Ca^2+^ increased activity by 31% and 17%, respectively, while K + and La3 + had negligible effects. In contrast, Zn^2+^, Ni^+^, Mn^2+^, Rb^+^, Co^2+^, Cu^2+^, Na^+^, and Mg^2+^ inhibited activity to varying extents, with Zn^2+^ causing more than 67% inhibition. At 50 mM, most metal ions except Na^+^ caused substantial inhibition. Notably, the inhibitory effect depended on the salt type; for example, CuCl_2_ caused total inactivation, while CuSO_4_ retained 15% activity. These results are presented in Table 1.

**Table 1:** Effect of metal ions on the activity of Recombinant 6xHis-SALD_AP_

Metal Ions	Metal Salts	Relative activity (%)	
		1 mM	50 mM
None	None	100	100
Ca^2+^	Calcium chloride	117	40
K^+^	Potassium chloride	101	84
Mg^2+^	Magnesium chloride	99	61
Na^+^	Sodium chloride	95	101
Cu^2+^	Copper (II) sulphate	51	15
Cu^2+^	Copper (II) chloride	50	0
Fe^2+^	Ferrous sulfate	131	0
La^3+^	Lanthanum (III) chloride	101	0
Co^2+^	Cobalt chloride	96	0
Rb^+^	Rubidium chloride	92	0
Mn^2+^	Manganese (II) chloride	88	0
Ni^+^	Nickel (II) chloride	87	0
Zn^2+^	Zinc sulphate	33	0

The activity of SALD_AP_ was assayed using 100 mM Tricine buffer (pH 8.0), 50 µM NAD^+^, 70 µM salicylaldehyde and 1 µM enzyme. Values reported are relative to the activity of the control (set as 100%)

The contrasting effects of CuCl₂ and CuSO₄ on SALD_AP_ activity can be explained by the different properties of chloride and sulphate anions. Chloride is monovalent and weakly hydrated, which facilitates the formation of reactive copper–chloride complexes (e.g., [CuCl₄]²⁻) and increases the effective availability of Cu²⁺ to interact with protein residues, leading to stronger inhibition. In contrast, sulphate is divalent and strongly hydrated, with weaker coordinating ability, which reduces the proportion of free Cu²⁺ available for inhibitory interactions [41]. These ion-specific effects are consistent with the Hofmeister series, which predicts that differences in hydration and binding properties influence protein stability and macromolecular interactions [42].

Taken together, these findings differ from previous reports on *Pseudomonas* SALDs [1, 17, 18], which showed different metal ion sensitivities. For example, calcium was detected in a *Pseudomonas* sp. strain C6 SALDH, where it was proposed to stabilise enzyme structure rather than enhance activity [18]. Our observation that Fe²⁺ enhanced SALD_AP_ activity is consistent with earlier work on *Pseudomonas* putida NahV and NahF, which also showed stimulation by Fe²⁺ [17]. However, unlike those enzymes, SALD_AP_ was additionally stimulated by Ca²⁺ and strongly inhibited by Zn²⁺, highlighting a distinct sensitivity profile compared with previously characterised bacterial SALDs. This diversity of responses is consistent with broader evidence that metals can act as structural stabilisers, activators, or inhibitors depending on the specific enzyme context [43, 44].

SALD_AP_ activity was also affected by organic solvents (Table 2). At low concentrations, ethanol and DMSO enhanced activity by ∼35% and 18%, respectively, while methanol and isopropanol had no effect. At moderate concentrations (10% v/v), activity decreased for all solvents, but higher concentrations (50% v/v) of solvents inhibited the enzyme, except DMSO, which retained more than 30% activity. These results are consistent with evidence that low concentrations of DMSO can stabilise protein structure and function [31].

**Table 2:** Effect of organic solvents, detergents and modulators on SALD_AP_ activity

Compound	Final Conc.	Relative activity (%)	Compound	Final Conc.	Relative activity (%)
Organic solvents (% v/v)					
Methanol	1	99.4 ± 0.7	Ethanol	1	134.6 ± 0.8
	10	68.0 ± 0.6		10	86.5 ± 1.0
	50	0.0 ± 0.0		50	0.0 ± 0.0
Isopropanol	1	99.5 ± 0.8	DMSO	1	118.1 ± 0.4
	10	82.1 ± 0.8		10	97.2 ± 0.8
	50	0.0 ± 0.0		50	33.4 ± 0.2
Detergents (% w/v)					
Triton X-100	1	107.6 ± 5.0	Tween-20	1	102.8 ± 7.5
	10	68.9 ± 6.1		10	71.4 ± 4.8
Enzyme inhibitors					
EDTA (µM)	10	115.8 ± 0.9	DTT (mM)	0.5	102.4 ± 0.6
	20	114.3 ± 0.7		1	101.4 ± 0.6
	50	111.4 ± 1.0		5	96.8 ± 0.7
	80	106.4 ± 1.1		10	95.3 ± 0.6
	100	104.2 ± 0.8		20	93.4 ± 0.5
	500	90.0 ± 0.6		50	77.2 ± 0.4

The activity of SALD_AP_ was assayed using 100 mM Tricine buffer (pH 8.0), 50 µM NAD^+^, 70 µM salicylaldehyde and 1 µM enzyme. Values reported are relative to the activity of the control (set as 100%) and are from triplicate measurements presented as mean ± SD

Detergents (Tween-20, Triton X-100) and reducing/chelating agents had minimal effects, except at their highest concentrations, where the detergents showed a 30% reduction in activity, while EDTA and DTT showed 10% and 23% reductions, respectively. The inability of EDTA to inhibit SALD_AP_ indicates the absence of metal ions that are reversibly coordinated within the active site of the enzyme. By contrast, the observed stimulation by exogenous Ca²⁺ and Fe²⁺ likely reflects indirect effects on enzyme conformation or stability rather than a requirement for catalytic metal cofactors. However, very high concentrations (50 mM) of chelators (EDTA and EGTA) have been shown to reduce the activity of SALDH [18].

Collectively, these findings demonstrate that SALD_AP_ is generally tolerant to a broad range of chemical environments but is inhibited by high levels of certain metals or solvents. This pattern likely reflects the chaotropic or kosmotropic effects of these compounds on protein structure and stability [45].

### The SALD_AP_ Has Broad Substrate Specificity and High Catalytic Efficiency for Aromatic Aldehydes

SALD_AP_ displayed activity towards both aliphatic and aromatic aldehydes, with notably higher activity against aromatic substrates (Table 3; Figure S3). Catalytic efficiency, assessed by specificity constant (*k*_*cat*_^*ap*^/*k*_*m*_), was greatest for aromatic aldehydes, with benzaldehyde being the preferred substrate. The *k*_*m*_ values for aromatics were in the low micromolar range (0.76–13.14 µM), indicating high affinity, whereas *k*_*m*_ values for aliphatic aldehydes were substantially higher (230–5697 µM). Although low *K*_*m*_ values for aromatic aldehydes suggest strong binding, *K*_*m*_ does not always directly equate to substrate affinity because it reflects both binding (*k*_*on*_, *k*_*off*_) and catalytic turnover (*k*_*cat*_) [46]. This is particularly relevant for SALD_AP_, where substrate inhibition was observed for benzaldehyde and 3-hydroxybenzaldehyde (Table S2), indicating that additional factors beyond simple binding contribute to the kinetic behaviour. Thus, while *K*_*m*_ provides a useful comparative parameter, catalytic efficiency (*k*_*cat*_^*ap*^*/K*_*m*_) is a more reliable descriptor of substrate preference in this system. SALD_AP_ thus binds and processes aromatic aldehydes much more efficiently than aliphatic ones.

**Table 3:** Substrate specificities and steady state kinetic parameters of 6xHis-SALD_AP_ for aromatic and aliphatic aldehydes

Substrate	K_m_ (µM)	V_max_ (µM s^− 1^)	K_cat_^ap^ (s^− 1^)	K_cat_^ap^/K_m_ 10^3^ (M^− 1^ s^− 1^)
2-Hydroxy-benzaldehyde (Salicylaldehyde)	13.14 ± 1.66	0.72 ± 0.03	48.0 ± 2.0	3700 ± 430
3-Hydroxy-benzaldehyde	0.99 ± 0.29	1.41 ± 0.08	94.3 ± 5.3	95,200 ± 27,300
Benzaldehyde	0.76 ± 0.29	1.81 ± 0.12	120.5 ± 8.0	158,500 ± 63,700
Cyclohexane carboxaldehyde	62.15 ± 7.65	4.24 ± 0.23	42.4 ± 2.3	700 ± 80
Propionaldehyde	5685.00 ± 937.40	3.28 ± 0.28	5.9 ± 0.5	1.03 ± 0.17
Butyraldehyde	4473.00 ± 411.50	7.53 ± 0.33	26.9 ± 1.2	6.01 ± 0.54
Isobutyraldehyde	5697.00 ± 367.00	6.63 ± 0.22	23.7 ± 0.8	4.16 ± 0.26
Crotonaldehyde	3319.00 ± 524.00	1.58 ± 0.11	5.6 ± 0.4	1.70 ± 0.26
Valeraldehyde	1450.00 ± 118.20	7.39 ± 0.24	26.4 ± 0.9	18.20 ± 1.40
Hexaldehyde	230.40 ± 57.60	5.97 ± 0.37	21.3 ± 1.3	92.50 ± 22.20
Heptaldehyde	504.60 ± 62.05	7.51 ± 0.44	26.8 ± 1.6	53.20 ± 6.40
Octaldehyde	469.10 ± 51.35	5.18 ± 0.26	51.8 ± 2.6	110 ± 12.00
Decanaldehyde	12,240 ± 298.20	5.33 ± 0.50	53.3 ± 5.0	43.50 ± 10.60

The activity of SALD_AP_ was assayed using varied enzyme and substrate concentrations in 100 mM Tricine buffer (pH 8.0) and 50 µM NAD^+^ at 25 °C. Values of Kinetic parameters reported are those returned from non-linear fitting using Eq. 1 and are shown as ± standard errors derived from this process. Refer to Table S2 and Figure S3 for additional kinetic data and individual plots, respectively. Enzyme purity was confirmed by SDS-PAGE, which showed a single band at the expected molecular weight (Figure S2b). Turnover numbers are reported as *k*_*cat*_^*ap*^, reflecting that values are based on total protein concentration measured by the Bradford assay

The substrate preference (benzaldehyde > 3-hydroxybenzaldehyde > salicylaldehyde) matches previous reports on *Pseudomonas* SALDs [1, 4, 18]. The activity of an enzyme towards an aromatic substrate depends on the size and the position of the substituent(s) attached to the aromatic ring, which in turn affects several factors that play crucial roles in activity. These factors include hydrogen bonding, hydrophobicity, electronic and steric effects [47, 48].

Turnover number (*k*_*cat*_^*ap*^) was highest for benzaldehyde (120.5 s^− 1^), while short-chain aliphatic aldehydes, propionaldehyde and crotonaldehyde exhibited much lower rates of 5.9 and 5.6 s^− 1^, respectively. This represents ∼20-fold lower *k*_*cat*_^*ap*^ than benzaldehyde. Long-chain aliphatic substrates showed intermediate turnover. For comparison, although *Pseudomonas* NahF and SALDH have higher affinity for benzaldehyde, their highest *k*_*cat*_^*ap*^ values were observed with salicylaldehyde [1, 18].

**Fig. 4:**
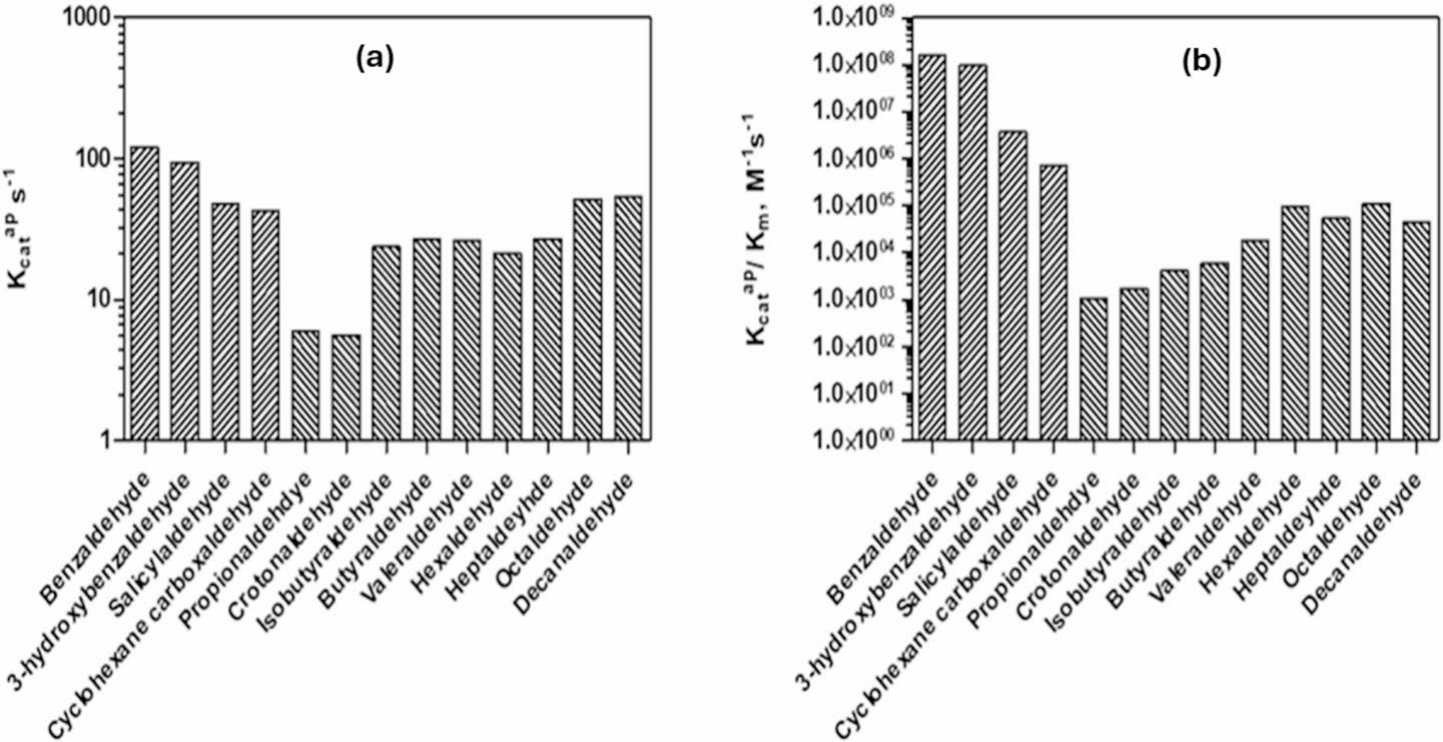
Logarithmic plots of (**a**) *K*_*cat*_^*ap*^ and (**b**) *K*_*cat*_^*ap*^*/k*_*m*_ for the oxidation of aromatic and aliphatic aldehydes by 6xHis-SALD_AP_ in the presence of 50 µM NAD^+^ at 25 °C

Overall, SALD_AP_ catalysed aromatic substrate oxidation with catalytic efficiencies (*k*_*cat*_^*ap*^*/k*_*m*_) exceeding 10^6^ M^− 1^ s^− 1^, markedly higher than for aliphatics. Among aliphatic aldehydes, activity generally increased with chain length up to C8–C10, though this was offset by reduced binding affinity. Short-chain substrates were the least efficient, medium-chain aldehydes showed intermediate performance, and long-chain aldehydes displayed higher turnover, but only modest efficiency compared to aromatics. Branched isobutyraldehyde exhibited similar kinetic parameters to its linear analogue, butyraldehyde, suggesting that C4 branching does not significantly affect catalysis. Thus, while chain elongation improves turnover, it does not overcome the inherent binding preference for aromatic substrates. To better visualise these trends, *K*_*cat*_^*ap*^, and *K*_*cat*_^*ap*^*/K*_*m*_ values for SALD_AP_ are presented graphically in Fig. 4.

Substrate inhibition was observed for some substrates (Table S2), especially benzaldehyde and 3-hydroxybenzaldehyde, a phenomenon also reported for other ALDHs [1, 18, 47, 49]. This may serve regulatory functions in metabolic pathways [50], though its mechanism remains unclear. Importantly, the marked preference for aromatic aldehydes is consistent with the ecological context of alpine soils, which are enriched in plant-derived aromatics such as lignin degradation products. By contrast, the modest activity towards aliphatic substrates may reflect a secondary or opportunistic role. These results suggest that SALD_AP_ is functionally specialised for aromatic catabolism in soil microbiomes, while retaining a broader substrate scope that may support metabolic flexibility under variable nutrient conditions.

### Ligands Stabilise SALD_AP_ against Heat Denaturation

Differential scanning fluorimetry (DSF) was employed to evaluate the thermal stability of SALD_AP_ and the effect of ligands (substrates and cofactors) on its melting temperature (T_m_). The untreated enzyme exhibited a T_m_ of ∼68 °C, with higher stability observed in the presence of both substrate and cofactor (Table 4). Aromatic aldehydes and NAD^+^, especially when combined, significantly increased the T_m_ (up to 71 °C), indicating that ligand binding stabilises SALD_AP_. The enzymes T_m_ were higher than those reported for yeast ALDHs [29, 51], suggesting greater intrinsic stability.

**Table 4:** Thermal stability of 6xHis-SALD_AP_ showing its melting temperatures (T_m_^o^C) upon interaction with different ligands

Aromatic Substrate/Cofactor	T_m_^o^C	Aliphatic Substrate/Cofactor	T_m_^o^C	Ligand/Cofactor	T_m_^o^C
Untreated	67.9 ± 0.2	Propionaldehyde	68.8 ± 0.3	Salicylaldehyde only	67.7 ± 0.3
NAD^+^	68.7 ± 0.3	Crotonaldehyde	68.8 ± 0.3	Calcium only	67.8 ± 0.2
Benzaldehyde	69.7 ± 0.4*	Butyraldehyde	68.9 ± 0.1*	NAD^+^ + Ca^2+^	68.5 ± 0.5
Salicylaldehyde	70.3 ± 0.4*	Isobutyraldehyde	69.2 ± 0.3*	NAD^+^ + Sal + Ca^2+^	69.6 ± 0.4*
3-hydroxybenzaldehyde	70.6 ± 0.4*	Valeraldehyde	69.3 ± 0.3*	DMSO only	66.7 ± 0.3*
4-hydroxybenzaldehyde	70.0 ± 0.3*	Hexaldehyde	70.9 ± 0.4*	DMSO + Calcium	67.3 ± 0.3
3,4-dihydroxybenzaldehyde	71.0 ± 0.0*	Heptaldehyde	70.3 ± 0.3*	NAD^+^ + DMSO	68.6 ± 0.1
Cyclohexane carboxaldehyde	71.0 ± 0.5*	Octaldehyde	69.7 ± 0.3*	NAD^+^ + DMSO + Calcium	68.8 ± 0.1
Vanillin	69.6 ± 0.4*	Decanaldehyde	69.6 ± 1.2*	NAD^+^ + DMSO + Ca^2+^ + Sal	69.9 ± 0.2*

The T_m_^o^C of SALD_AP_ bound to aldehyde substrates and cofactor were measured in the presence of 2 mM substrates and 1.5 mM NAD^+^. The T_m_^o^C of the enzyme bound to cofactor only was measured in the presence of 1.5 mM NAD^+^. All experiments were carried out using 6 µM enzyme. The values indicate means of triplicate measurements ± standard deviation. All results were compared with the T_m_^o^C of the untreated enzyme (control) for statistical significance using one-way ANOVA and Dunnett’s multiple comparison post-test. (*) indicates a statistically significant difference (*p* < 0.05) between the test and the control

Neither NAD⁺ nor salicylaldehyde alone significantly stabilised the enzyme, highlighting that both are required for maximal thermal stability. Enhanced stability was also more pronounced with aromatic aldehydes and longer-chain aliphatic substrates; short-chain aliphatic aldehydes provided little or no stabilisation, paralleling trends in catalytic efficiency (Table 3; Figure S3). Substrates that did not stabilise SALD_AP_ (propionaldehyde, crotonaldehyde) also showed low activity and weak binding.

Calcium, which increased activity in solution assays, was specifically chosen for DSF analysis because calcium has been reported at stoichiometric levels (1.8 mol/mol enzyme) in the SALD from *Pseudomonas* sp. strain C6, where it was suggested to contribute to structural stability [18]. In contrast, Fe²⁺, although it also enhanced the activity of SALD_AP_, was not tested in DSF because it is redox-active and could interfere with fluorescence-based stability measurements through non-specific oxidative effects. In SALD_AP_, calcium did not significantly alter the enzyme’s T_m_, either alone or in combination with NAD⁺ and/or substrate. Low concentrations of DMSO (1%) significantly (*p* < 0.05) decreased T_m_ by about 1.2 °C; this destabilisation was partially reversed by calcium and completely masked by NAD⁺ or substrate. This is consistent with reports that DMSO can destabilise certain protein-ligand complexes [52]. Although the effect of other solvents (methanol, ethanol, and isopropanol) on the activity of SALD_AP_ was tested, DSF analysis was performed only with DMSO, as this was the sole solvent used in substrate assays to increase aldehyde solubility; other solvents were not investigated further.

DSF melting curves revealed a biphasic pattern with two peaks (∼35 °C and ∼68 °C) in the untreated enzyme (Figure S4), suggesting the presence of multiple structural states rather than contamination, as confirmed by protein homogeneity via SDS-PAGE (Figure S2b). The lower T_m_ transition may correspond to partial unfolding or oligomer dissociation, while the higher T_m_ peak represents unfolding of the intact holoenzyme. Ligand binding shifted the equilibrium towards the higher T_m_ state: NAD⁺ alone provided modest stabilisation, whereas aromatic aldehydes alone had little effect. In contrast, when NAD⁺ and an aromatic substrate were combined, the lower transition was suppressed and the higher T_m_ peak was enhanced and shifted upwards (to ∼70–71 °C), consistent with cooperative stabilisation of the enzyme–cofactor–substrate complex. This biphasic behaviour, similar to that observed for coenzyme A binding to WcbI from *Burkholderia pseudomallei* [53], therefore likely reflects distinct unfolding transitions of SALD_AP_ and its ligand-bound states. In contrast, yeast ALDHs exhibit monophasic melting under comparable conditions [29, 51], possibly due to differences in subunit organisation.

Overall, the SALD_AP_ melting pattern shows that when its cofactor and/or substrates bind, the protein favours the peak to the right, signifying denaturation at the ligand-bound melting temperature (Figures S4a, b, c, and d), conferring significant thermal stability to SALD_AP_. Conversely, in the presence of inhibitors such as DMSO, the ligand-free melting temperature to the left is favoured (Figure S4e).

### Molecular Docking Elucidates Substrate Specificity and Catalytic Mechanism in SALD_AP_ and its Structural Analogue NahF

The crystal structure of SALD_AP_, previously resolved by our group, revealed high structural similarity to *Pseudomonas putida* NahF, despite low sequence identity [19]. Being the only two SALD crystal structures in the Protein Data Bank (PDB), we performed molecular docking with 17 aldehyde substrates (aromatic and aliphatic) to elucidate substrate interactions and specificity.

Docking results (Table 5) showed that aromatic substrates bind more tightly to both SALD_AP_ and NahF, as reflected by lower binding energies (more negative ΔG) and dissociation constants (K_d_), compared to short-chain aliphatic substrates. The binding affinity for aliphatic substrates improved with increasing chain length. NahF exhibited generally higher affinity for aromatic substrates than SALD_AP_, while affinities for aliphatic substrates were comparable between the two enzymes.

**Table 5:** Summary of in Silico Docking results with the crystal structures of SALD_AP_ and NahF

	SALD_AP_ (PDB: 6QHN)				NahF (PDB: 4JZ6)			
Substrate	ΔG (Kcal/mol)	K_D_ (µM)	Interacting residue	H-bond (Å)	ΔG (Kcal/mol)	K_D_ (µM)	Interacting residue	H-bond (Å)
Benzaldehyde	−5.2	154.2	ASN137	2.2	−5.3	130.2	CYS284	2.0
Salicylaldehyde	−5.4	110.0	ASN137	2.2	−5.4	110.0	CYS284	2.0
3-hydroxybenzaldehyde	−5.2	154.2	ASN137	1.9	−6.1	33.8	CYS284	1.9
4-hydroxybenzaldehyde	−5.2	154.2	ASN137	2.0	−5.7	66.3	CYS284	2.0
3,4-dihydroxybenzaldehyde	−5.1	182.5	ASN137	1.9	−5.7	66.3	CYS284	2.2
Vanillin	−5.3	130.2	ASN137	2.0	−6.0	40.0	CYS284	2.1
2-naphthaldehyde	−6.6	14.5	ASN137	1.8	−7.1	6.2	ARG157	2.4
Cyclohexane carboxaldehyde	−5.0	216.1	ASN137	2.2	−5.2	154.2	SER208	2.4
Propionaldehyde	−2.9	7482.9	ASN137	2.2	−2.9	7482.9	CYS284	2.0
Butyraldehyde	−3.3	3809.2	ASN137	2.2	−3.4	3217.6	ARG157	2.4
Isobutyraldehyde	−3.4	3217.6	ASN137	2.3	−3.4	3217.6	-	-
Crotonaldehyde	−3.3	3809.2	ASN137	2.2	−3.6	2295.7	CYS284	2.0
Valeraldehyde	−3.7	1939.1	ASN137	2.2	−3.8	1637.9	CYS284	2.0
Hexaldehyde	−4.1	987.1	ASN137	2.2	−4.1	987.1	CYS284	2.1
Heptaldehyde	−4.4	594.9	ASN137	2.2	−4.3	704.3	CYS284	2.1
Octanaldehyde	−4.7	358.5	ASN137	2.2	−4.7	358.5	ARG157	2.4
Decanaldehyde	−5.1	182.5	ASN137	2.1	−5.0	216.1	CYS284	2.7

ΔG is the binding energy

K_D_ is the substrate dissociation constant, and

H-bond is the hydrogen bond distance between the substrate substituent and the interacting residue

Interestingly, for most substrates, SALD_AP_ formed a key hydrogen bond between the aldehyde group and ASN137, whereas NahF typically interacted via CYS284. The different positioning of the substrate relative to active-site residues suggests distinct mechanistic strategies, despite structural similarity (see Figure S5).

Structural features underlying substrate specificity include the presence of an “aromatic box” [54]. In SALD_AP_, Ala138 and Phe433 fulfil this role, which is more consistent with a catalytic than a binding function. Additionally, the presence of a less bulky residue (Ile141 in SALD_AP_, Val153 in NahF) at the position analogous to Met177 in *S. cerevisiae* Ald6p likely enables accommodation and turnover of aromatic and cyclic substrates, as supported by mutagenesis studies in yeast ALDHs [29].

Overall, the docking analysis demonstrates that both SALD_AP_ and NahF preferentially bind and catalyse aromatic substrates. However, they use different active site residues to engage substrates, potentially reflecting mechanistic divergence within this enzyme family.

### Proposed Catalytic Mechanism of SALD_AP_

The catalytic mechanism of ALDHs typically involves hydride transfer from the substrate aldehyde to NAD^+^, via a tetrahedral intermediate stabilised by an invariant catalytic cysteine. Upon cofactor binding, this cysteine forms a thiohemiacetal intermediate with the substrate. Hydride transfer yields a thioacyl-enzyme and NADH, after which a conserved glutamate activates a water molecule to hydrolyse the thioester, regenerating the cysteine and releasing the carboxylic acid product [1, 8, 55]. Numerous mutagenesis studies support the central role of the invariant cysteine as the catalytic thiol [56–59].

In NahF, the proposed mechanism follows this canonical pathway, with CYS-284 acting as the catalytic thiol and ARG-157 aiding in the positioning of the nucleophilic water molecule [1]. Our docking studies support this model for NahF. Structural alignment further shows that SALD_AP_ and NahF share high overall similarity (rmsd 1.4 Å across 466 aligned residues), indicating that their active site architectures, including pocket volume, are broadly comparable. However, for SALD_AP_, docking and structural analysis reveal that substrate binding is primarily mediated by ASN-137, a strictly conserved residue which lies close to the catalytic cysteine and NAD^+^ [9, 60].

**Fig. 5:**
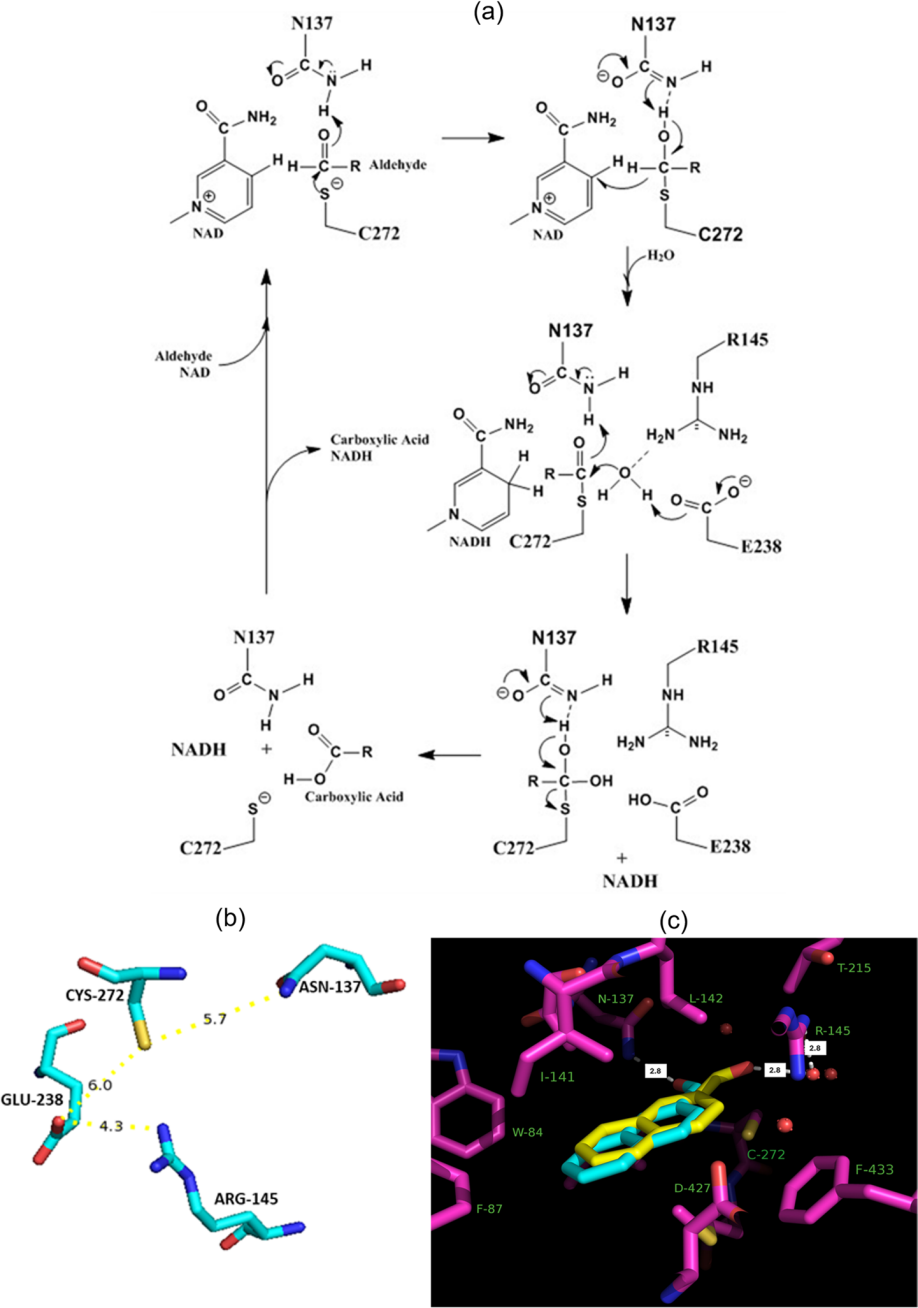
(**a**) Proposed reaction mechanism of novel SALD_AP_ based on its tertiary structure, molecular docking of various aldehydes into its catalytic site, the strict conservation of N137 in ALDHs and the potential role of R145. (**b**) The distances (Å) showing the proximity of the four amino acid residues in the active site of SALD_AP_ proposed to be involved in catalysis. (**c**) The electron density as seen in the active site of SALD_AP_ after refinement using 2-naphthaldehyde as a ligand, in two conformations (yellow and cyan carbons with occupancies of 0.4 and 0.6, respectively)

Based on our data, we propose a catalytic mechanism for SALD_AP_ involving ASN-137, ARG-145, GLU-238 and CYS-272 (Fig. 5a and b); the bolded residues are strictly conserved across ALDHs. Electron density maps and docking place the substrate carbonyl oxygen near ASN-137, supporting its central role in catalysis (Fig. 5c).

Upon cofactor binding, the catalytic cysteine becomes activated. Furthermore, aldehyde substrate binding reduces active site solvation, thereby increasing catalytic thiol nucleophilicity [59]. The thiol group attacks the substrate carbonyl (I), forming a transient tetrahedral intermediate. Stabilised by H-bonding between the ASN-137 side chain amide and the substrate’s carbonyl oxygen. A hydride transfer to NAD^+^ occurs (II), forming NADH and a thioester intermediate (III). The thioester is positioned and ready for base catalysis. GLU-238 activates a water molecule for nucleophilic attack on the thioester, generating a second, transient tetrahedral intermediate (IV). The nucleophilic water molecule is well-positioned by ARG-145, as clearly seen in the electron density map (Fig. 5c). The weak intermediate collapses to yield the product and regenerate the thiolate (V). Once the product dissociates, the regenerated enzyme becomes ready for another turnover.

While broadly consistent with the established ALDH paradigm, this mechanism uniquely highlights ASN-137’s key role in substrate binding and stabilisation and identifies ARG-145 as critical for water activation and positioning—roles not emphasised in previous models [60]. The spatial arrangement and proximity of these four active-site residues (Fig. 5b) provide a strong mechanistic framework for SALD_AP_ catalysis. While the proposed mechanism is strongly supported by structural alignment and docking analyses, experimental validation through site-directed mutagenesis of residues such as Asn-137, Arg-145, and Glu-238 will be required to confirm their precise functional roles. These studies are currently in progress and will provide critical evidence to strengthen the mechanistic model.

## Conclusion

In this study, we provide the first comprehensive biochemical and mechanistic characterisation of a salicylaldehyde dehydrogenase (SALD_AP_) derived from an alpine soil metagenome. Building on our previous structural report [19], we show that SALD_AP_ represents the first experimentally characterised alphaproteobacterial SALD, with strict NAD⁺ dependence, high catalytic efficiency for aromatic aldehydes, and distinct tolerance to diverse chemical environments. Detailed structural, biochemical, and molecular docking analyses reveal that SALD_AP_ exhibits a unique substrate-binding mode and a distinct catalytic mechanism centred on a strictly conserved asparagine residue, ASN-137, in concert with ARG-145, GLU-238, and CYS-272, which expands the mechanistic landscape of the ALDH superfamily. By linking structural features to substrate specificity and stability, this work establishes a new paradigm for aromatic aldehyde oxidation and highlights the ecological role of SALD_AP_ in alpine soil microbiomes. These findings provide a foundation for future engineering of ALDHs in biocatalysis, environmental detoxification, and synthetic biology applications.

## Supplementary Information

Below is the link to the electronic supplementary material.

ESM 1 (DOCX 1.62 MB)

## Data Availability

All experimental data and other results supporting the findings of this study are presented in the manuscript and supplementary files. Metagenomes and assembled sequences are available in the NCBI database under the Bioproject code and accession number specified in the manuscript.
