# Supplementary material for: A Novel Salicylaldehyde Dehydrogenase from Alpine Soil Metagenome Reveals a Unique Catalytic Mechanism
Source: Appl Biochem Biotechnol. 2025 Nov 3;197(12):8247–69. doi: 10.1007/s12010-025-05445-4 (PMC12718228; doi:10.1007/s12010-025-05445-4)
Supplement: Supplementary file 1 — (DOCX 1.62 MB) [file 12010_2025_5445_MOESM1_ESM.docx]

**A Novel Salicylaldehyde Dehydrogenase from Alpine Soil Metagenome Reveals a Unique Catalytic Mechanism**

Shamsudeen Umar Dandare^1,2^, Ibrahim Aliyu Dabai^1,3^, Deepak Kumaresan^1,4^, Christopher C.R. Allen^1^

1 School of Biological Sciences, Queen’s University Belfast, BT9 5DL, UK

2 Department of Biochemistry and Molecular Biology, Usmanu Danfodiyo University, Sokoto, Nigeria.

3 Agri-Environment Branch, Agri-Food and Biosciences Institute, Belfast, BT9 5PX, UK

4 School of Biosciences, University of Birmingham, Birmingham, B15 2TT, UK

Correspondence to [s.dandare@qub.ac.uk](mailto:s.dandare@qub.ac.uk); [c.allen@qub.ac.uk](mailto:c.allen@qub.ac.uk)

**Table S1:** Summary of contigs and ORFs of assembled aldehyde dehydrogenases

| Contigs | Nucleotides | Amino acids in ORF | Start and Stop codons | NCBI BLAST Identity |
| --- | --- | --- | --- | --- |
| 1 | 1917 | 399 | Start only | Aldehyde dehydrogenase 60% |
| 2 | 1528 | 470 | Start and stop | Salicylaldehyde dehydrogenase 82% |


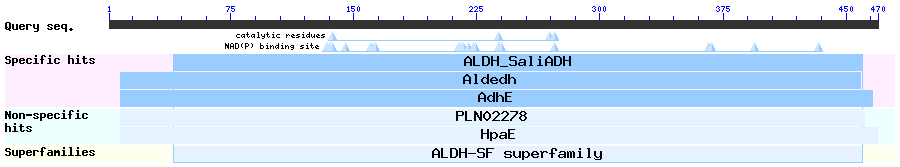


**Figure S1:** The NCBI graphical summary of the putative conserved domains of assembled salicylaldehyde dehydrogenase from alpine soil metagenome (SALD_AP_).


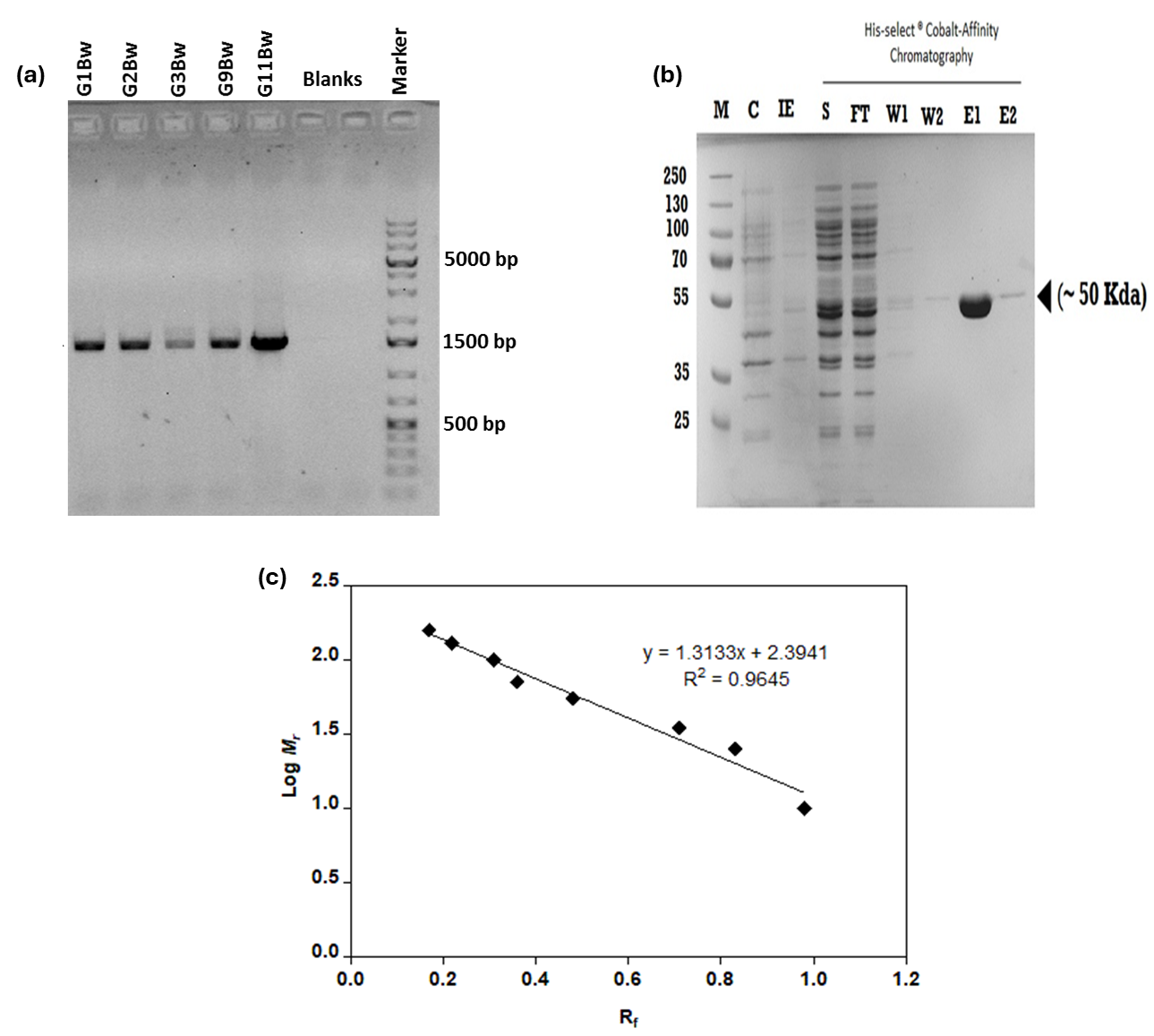


**Figure S2 (a)** 1% agarose gels showing specific SALD_AP_ genes obtained from PCR amplification of alpine metagenomes from the Bw horizon of different sample sites. **Marker:** 1 Kb plus GeneRuler (Thermo-Fischer Scientific, USA). **(b)** SDS-PAGE expression and purification of novel SALD_AP_ using Co^2+^-CMA affinity resin. M: Molecular weight markers (kDa); C: Total cell extract before induction; IE: Induced expression; S: Soluble lysate; FT: Flow-through; W1 & W2: First and second affinity column washes; E1 & E2: First and second protein elutions. **(c)** Calibration curve for SDS-PAGE broad-range molecular weight markers (Thermoscientific). For each marker, the log *M_r_* was plotted against the R_f_ value (ratio of the distance travelled by the protein to the distance travelled by the migration front). A total of eight proteins spanning a broad molecular weight range of 10-250 kDa were used.

**Table S2** Substrate specificities and steady state kinetic parameters of 6xHis-SALD_AP_ for aromatic and aliphatic aldehydes

| **Substrate** | ***K_m_* (µM)** | ***V_max_* (µM s^-1^)** | **[E]_o_ 10^-3^ (µM)** | ***K_cat_*^ap^ (s^-1^)** | ***K_cat_*^ap^/*K_m_*  10^3^**  **(M^-1^ s^-1^)** | ***K_i_*  (µM)** |
| --- | --- | --- | --- | --- | --- | --- |
| 2-Hydroxy-benzaldehyde (Salicylaldehyde) | 13.14 ± 1.66 | 0.72 ± 0.03 | 15.0 | 48.0 ± 2.0 | 3700 ± 430 | NIO |
| 3-Hydroxy-benzaldehyde | 0.99 ± 0.29 | 1.41 ± 0.08 | 15.0 | 94.3 ± 5.3 | 95200 ± 27300 | 53.03 ± 44.40 |
| Benzaldehyde | 0.76 ± 0.29 | 1.81 ± 0.12 | 15.0 | 120.5 ± 8.0 | 158500 ± 63700 | 5.02 ± 1.84 |
| Cyclohexane carboxaldehyde | 62.15 ± 7.65 | 4.24 ± 0.23 | 100.0 | 42.4 ± 2.3 | 700 ± 80 | NIO |
| Propionaldehyde | 5685.00 ± 937.40 | 3.28 ± 0.28 | 560.0 | 5.9 ± 0.5 | 1.03 ± 0.17 | NIO |
| Butyraldehyde | 4473.00 ± 411.50 | 7.53 ± 0.33 | 280.0 | 26.9 ± 1.2 | 6.01 ± 0.54 | NIO |
| Isobutyraldehyde | 5697.00 ± 367.00 | 6.63 ± 0.22 | 280.0 | 23.7 ± 0.8 | 4.16 ± 0.26 | NIO |
| Crotonaldehyde | 3319.00 ± 524.00 | 1.58 ± 0.11 | 280.0 | 5.6 ± 0.4 | 1.70 ± 0.26 | NIO |
| Valeraldehyde | 1450.00 ± 118.20 | 7.39 ± 0.24 | 280.0 | 26.4 ± 0.9 | 18.2 ± 1.40 | NIO |
| Hexaldehyde | 230.40 ± 57.60 | 5.97 ± 0.37 | 280.0 | 21.3 ± 1.3 | 92.5 ± 22.2 | 5411 ± 1486 |
| Heptaldehyde | 504.60 ± 62.05 | 7.51 ± 0.44 | 280.0 | 26.8 ± 1.6 | 53.2 ± 6.4 | NIO |
| Octaldehyde | 469.10 ± 51.35 | 5.18 ± 0.26 | 100.0 | 51.8 ± 2.6 | 110 ± 12.0 | NIO |
| Decanaldehyde | 1224.00 ± 298.20 | 5.33 ± 0.50 | 100.0 | 53.3 ± 5.0 | 43.5 ± 10.6 | 770.7 ± 505.8 |

The activity of SALD_AP_ was assayed using varied enzyme and substrate concentrations in 100 mM Tricine buffer (pH 8.0) and 50 µM NAD^+^ at 25 ^o^C. Values of Kinetic parameters reported are those returned from non-linear fitting and are shown as ± standard errors derived from this process. NIO: No inhibition observed.


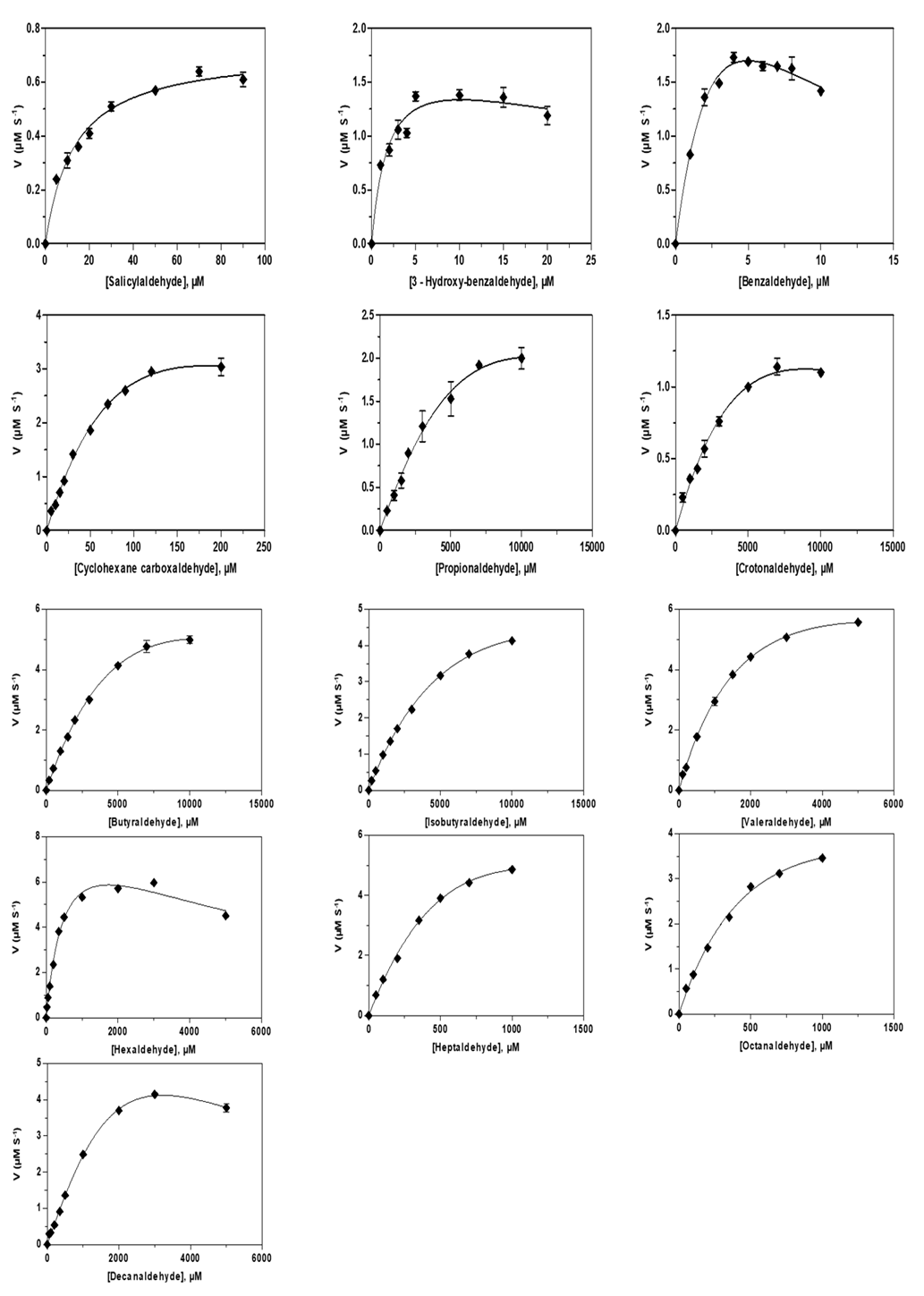


**Figure S3** Initial rates as a function of substrate concentration for the oxidation of aromatic and aliphatic aldehydes catalysed by 6xHis-SALD_AP_ in the presence of 50 µM NAD^+^ at 25.0 ^o^C, and 100 mM Tricine pH 8.0. Lines drawn through the experimental data are fits to Equations 1 or 2 with the kinetic parameters and the inbition constant (K_i_) for those substrates presented in Table S2 above.


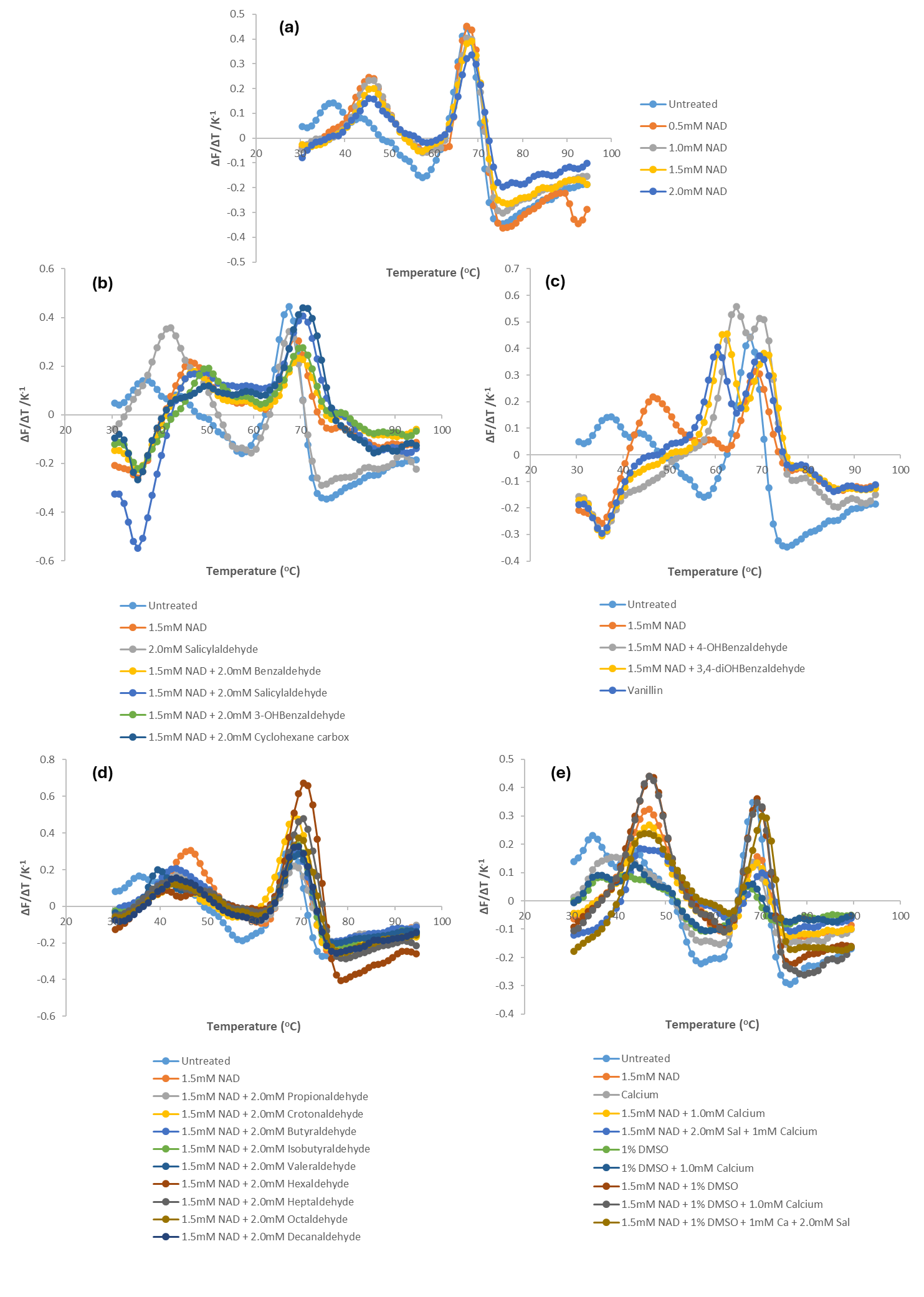


**Figure S4** shows the melting curves of SALD_AP_ showing changes in melting temperatures upon binding of the enzyme with **(a)** different concentrations of NAD^+^, **(b)** and **(c)** optimum concentrations of NAD+ and aromatic substrates, **(d)** optimum concentrations of NAD+ and aliphatic substrates, and **(e)** some ligands and their various combinations.


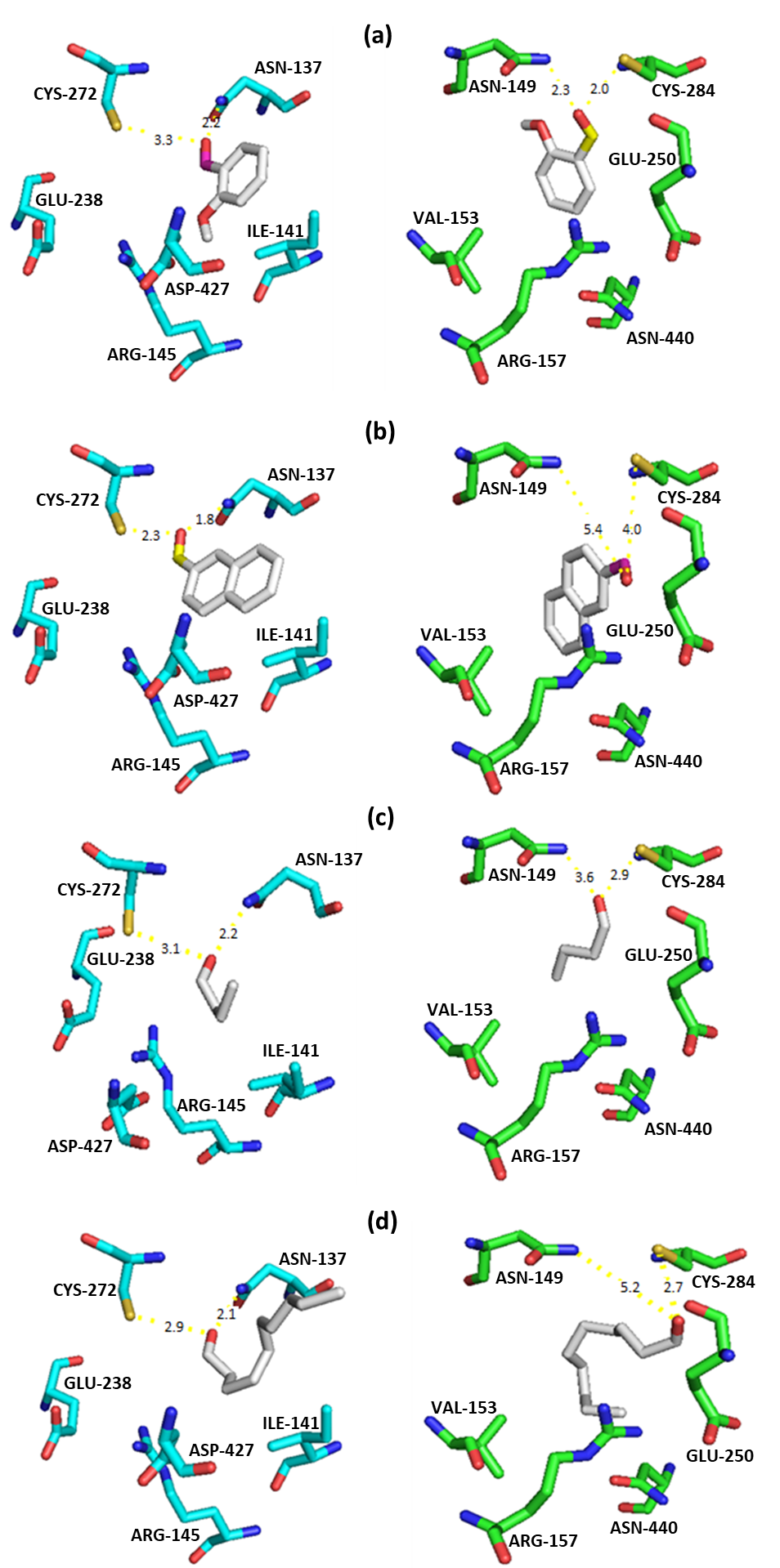


**Figure S5** Representative molecular docking of SALD_AP_ (cyan) and NahF (green) with **(a)** Salicylaldehyde, **(b)** 2-naphthaldehyde, **(c)** butyraldehyde, **(d)** decanaldehyde. The carbon chains of the substrates are in ash surrounded by amino acid residues of the substrate binding pocket. The yellow lines indicate the hydrogen bonds, and the numbers show the distance in Armstrong (Å).
